# An adult autosomal recessive chronic granulomatous disease patient with pulmonary Aspergillus terreus infection

**DOI:** 10.1186/s12879-018-3451-8

**Published:** 2018-11-08

**Authors:** Esmaeil Mortaz, Somayeh Sarhifynia, Majid Marjani, Afshin Moniri, Davood Mansouri, Payam Mehrian, Karin van Leeuwen, Dirk Roos, Johan Garssen, Ian M. Adcock, Payam Tabarsi

**Affiliations:** 1 Clinical Tuberculosis and Epidemiology Research Centre, National Research Institute for Tuberculosis and Lung Disease (NRITLD), Shahid Beheshti University of Medical Sciences, Tehran, Iran; 2 Department of Immunology, School of Medicine, Shahid Beheshti University of Medical Sciences, Tehran, Iran; 3 Division of Pharmacology, Utrecht Institute for Pharmaceutical Sciences, Faculty of Science, Utrecht University, Utrecht, The Netherlands; 4 Sanquin Research and Landsteiner Laboratory, Academic Medical Centre, University of Amsterdam, Amsterdam, The Netherlands; 5 Nutricia Research Centre for Specialized Nutrition, Utrecht, The Netherlands; 6 Priority Research Centre for Healthy Lungs, Hunter Medical Research Institute, The University of Newcastle, Newcastle, New South Wales Australia; 7 Cell and Molecular Biology Group, Airways Disease Section, National Heart and Lung Institute, Imperial College, London, UK

**Keywords:** CGD, Aspergillus terreus, Pulmonary infection

## Abstract

**Background:**

Genetic mutations that reduce intracellular superoxide production by granulocytes causes chronic granulomatous disease (CGD). These patients suffer from frequent and severe bacterial and fungal infections throughout their early life. Diagnosis is usually made in the first 2 years of life but is sometimes only diagnosed when the patient is an adult although they may have suffered from symptoms since childhood.

**Case presentation:**

A 26-year-old man was referred with weight loss, fever, hepatosplenomegaly and coughing. He had previously been diagnosed with lymphadenopathy in the neck at age 8 and prescribed anti-tuberculosis treatment. A chest radiograph revealed extensive right-sided consolidation along with smaller foci of consolidation in the left lung. On admission to hospital he had respiratory problems with fever. Laboratory investigations including dihydrorhodamine-123 (DHR) tests and mutational analysis indicated CGD. Stimulation of his isolated peripheral blood neutrophils (PMN) with phorbol 12-myristate 13-acetate (PMA) produced low, subnormal levels of reactive oxygen species (ROS). *Aspergillus terreus* was isolated from bronchoalveolar lavage (BAL) fluid and sequenced.

**Conclusions:**

We describe, for the first time, the presence of pulmonary *A. terreus* infection in an adult autosomal CGD patient on long-term corticosteroid treatment. The combination of the molecular characterization of the inherited CGD and the sequencing of fungal DNA has allowed the identification of the disease-causing agent and the optimal treatment to be given as a consequence.

## Background

Chronic granulomatous disease (CGD) is a rare inherited primary immune deficiency disorder [1–3]. It presents as life-threatening recurrent fungal and bacterial infections of the skin, lungs and bones with associated chronic inflammation or granulomas. As its name suggests, granulocytes (neutrophils and eosinophils) along with monocytes and macrophages are the major cellular targets. These cells have reduced phagocysis due to defective generation of reactive oxygen species (ROS) which are required to kill selective types of fungi and bacteria [1–3]. CGD is generally diagnosed in infancy or early childhood [4, 5].

Genetic defects in the nicotinamide adenine dinucleotide phosphate (NAPDH) oxidase complex can cause CGD. The 5 structural components that are mutated are the 3 cytoplasmatic components p47phox, p67phox and p40phox and the 2 membrane-associated glycoproteins gp91phox (phagocyte oxidase) and p22phox [6]. As a result, cells cannot eliminate infectious agents due to an inability to produce high levels of ROS. Subsequently, infections such as pneumonia, lymphadenitis, cutaneous and hepatic abscesses, osteomyelitis and septicemia are frequently observed and generally severe in nature [7]. The most commonly found infectious agents include Aspergillus species, Burkholderia cepacia complex, Candida, enteric gram-negative bacteria, Mycobacterium tuberculosis, marcescens and *Staphylococcus aureus* [8]. In addition, Aspergillus species such as *A. fumigatus*, *A. flavus* and *A. nidulans* are commonly isolated from CGD patients with fungal spondylodiscitis [9].

We describe here a case of autosomal CGD diagnosed in a 28-year-old male. He initially presented with granulomatous disease mimicking sarcoidosis and was treated with corticosteroids for 12 years. On admission to hospital he had respiratory problems with fever, and laboratory investigations including dihydrorhodamine-123 (DHR) tests [10] and mutational analysis indicated CGD. Aspergillus terreus was isolated from bronchoalveolar lavage (BAL) fluid and its presence was confirmed by sequencing.

## Case presentation

A 26-year-old man with a history of consanguineous parents (cousins) was referred with weight loss, fever, hepatosplenomegaly and coughing. He had previously been diagnosed with lymphadenopathy in the neck at age 8 and prescribed anti-tuberculosis treatment. At 12-years of age he was diagnosed with pulmonary sarcoidosis and corticosteroid treatment was initiated.

**Fig. 1:**
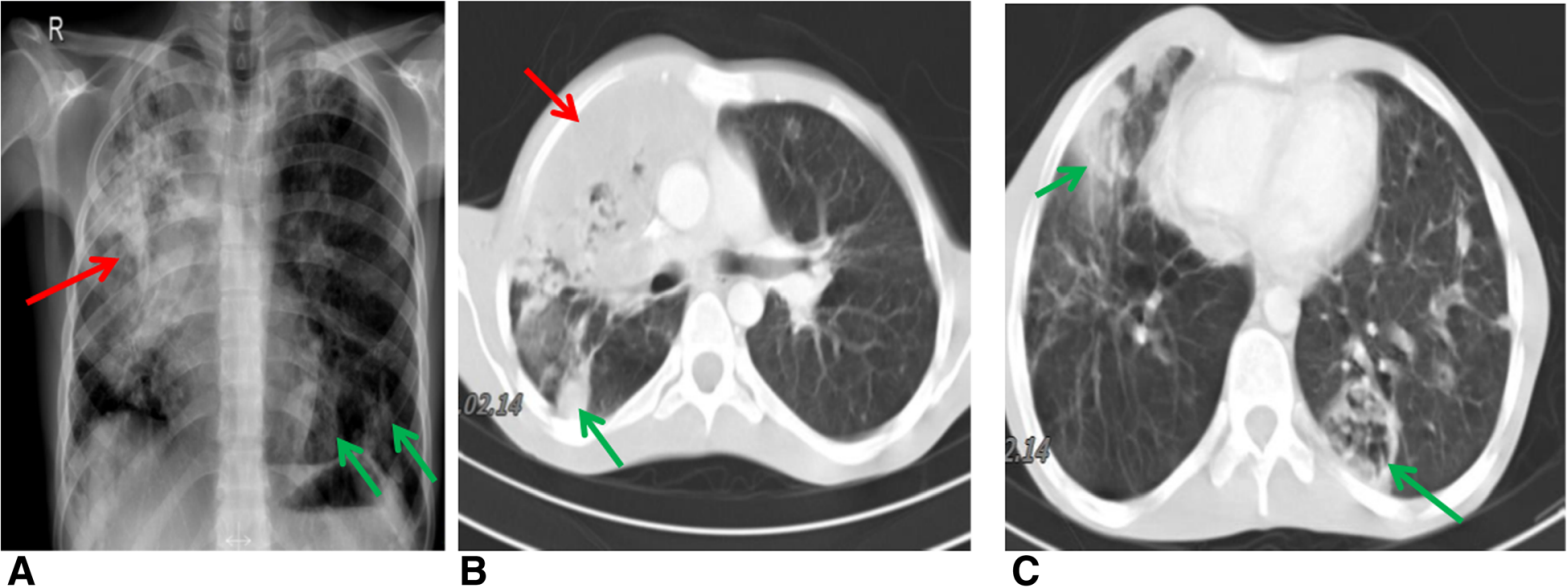
Lung imaging. **a**. There is extensive consolidation in the right lung (red arrow). Scattered patches of consolidation in the left lung are also seen (green arrow). Chest CT scans show the lung window at the level of the pulmonary artery (**b**) and heart (**c**). Note extensive consolidation in the right upper lobe (red arrow **b**). There are also smaller patches of consolidation in the right lower and middle lobes and in the left lung (green arrows **b** and **c**)

On examination on the day of admission to our hospital the patient was pale with low-grade pyrexia (37.5 °C). Cardiovascular examinations were normal but he had cervical lymphadenopathy. A chest radiograph revealed extensive right-sided consolidation along with smaller foci of consolidation in left lung (Fig. 1a). Crackles were heard on the left side.

Moxifloxacin treatment for 2 weeks did not alleviate his cough, hypoxia or night sweats and fevers of up to 40 °C. Thoracic computed tomography demonstrated mediastinal lymphadenopathy and bilateral consolidation that was greater in the right lung. Non-specific inflammation was shown in a lung biopsy (Fig. 1b and c). Full blood counts were normal and liver function tests and autoimmune and virology screens were negative.

**Fig. 2:**
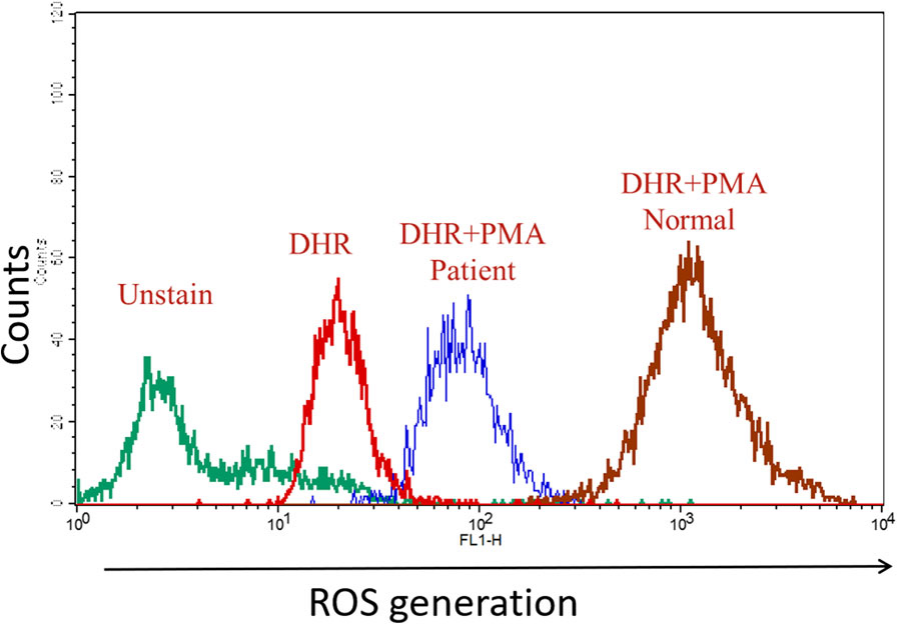
Dihydrorhodamine-1,2,3 (DHR) analysis of reactive oxygen species generation by peripheral blood neutrophils. Patient and healthy control cells were incubated with DHR (375 ng/ml), with or without PMA (100 ng/ml), and ROS generation was assayed by FACS analysis. The mean fluorescent intensity (MFI) of the following groups are indicated in the figure: Red lines represent cells from unstimulated healthy controls and patient cells, blue line represents PMA-stimulated patient cells, and grey line represents and PMA-stimulated healthy control cells

Whole blood was examined with specific laboratory tests for neutrophil NADPH oxidase activity i.e. nitroblue tetrazolium (NBT) [10] and DHR tests [10–14]. Low, subnormal levels of ROS were produced following stimulation of purified peripheral blood neutrophils (PMN) with phorbol 12-myristate 13-acetate (PMA) (Fig. 2).

**Fig. 3:**
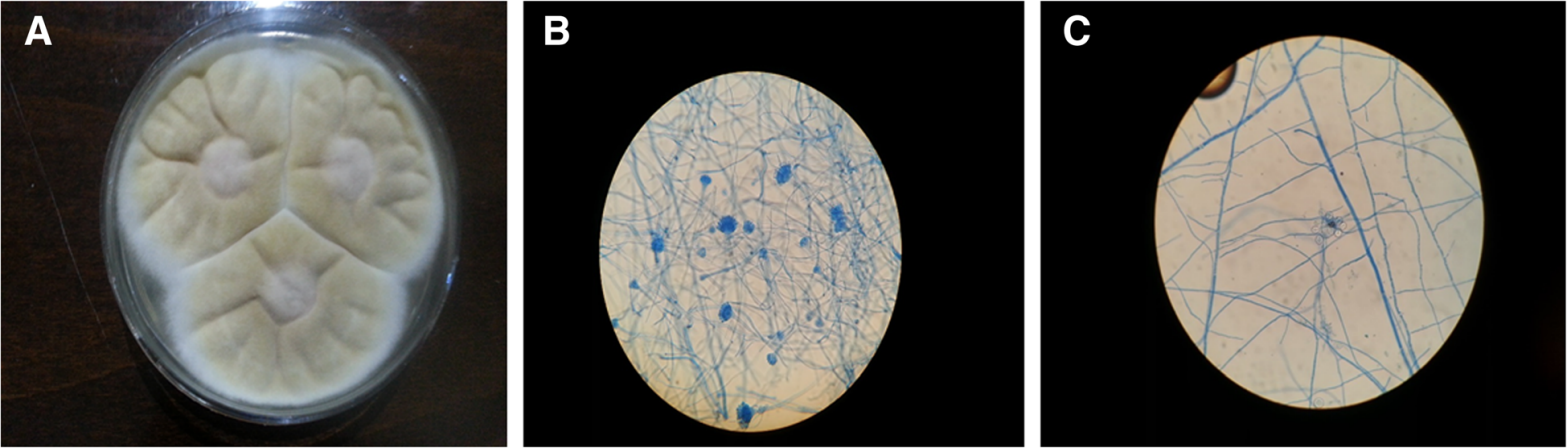
Microbiological culture of lung fungal species obtained by bronchoscopy. **a** 7-day-old culture on sabouraud dextrose agar at 30 °C shows light yellow to brownish colonies. **b** Fungal growth after 12 days sterile culture on potato dextrose agar (PDA) plates for sporulation and identification, and LPCB mounting slide showing details of hyphae and the accessory conidia. **c** Septate and hyaline hyphae with biseriate phialides extending from the upper portion of the vesicle and covering 2/3 of the plate

BAL and serum galactomannan (GAM) tests were negative but the BAL sample was sent for microbiological analysis. 48-72 h culture of the BAL sample on sabouraud dextrose agar at 30 °C resulted in the appearance of smooth light yellow powdery colonies that became darker over time (Fig. 3a). Lactophenol cotton blue (LPCB) mounting medium slide culture was performed for microscopic species identification (Fig. 3b and c). Microscopic analysis revealed septate and hyaline hyphae with biseriate phialides extending from the upper portion of the vesicle and covering 2/3 of the plate. Hyaline, globose or oval and thick-walled chlamydoconidia were also seen (Fig. 3b and c). The isolates were identified by phenotypic (macroscopic and microscopic) characteristics as *Aspergillus terreus,* and this identification was confirmed by DNA sequencing.

**Fig. 4:**
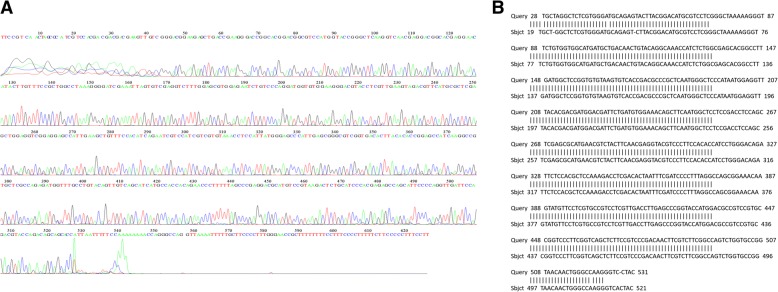
**a** Partial sequence of the putative invertase gene from *Aspergillus terreus* isolated from the patient. This is compared to the sequence of the ‘standard’ *A. terreus* sp. (**b**)

The fungal culture was disrupted with glass beads in a grinder and DNA was extracted with phenol chloroform. DNA was suspended in 50 μl of double distilled water and stored at − 20 °C for future use [1, 2]. The beta tubulin gene was amplified with forward (Bta2a: 5′-GGTAACCAAATCGGTGCTGCTTTC-3′) and reverse (Bta2b 5′-ACCCTCAGTGTAGTGACCCTTGGC-3′) primers and sequenced. The DNA sequence results were compared against the NCBI Genebank database, which showed a 99% similarity with an *Aspergillus terreus* isolate in the Gene Bank fungal library with accession no 1168 [15, 16] (Fig. 4).

Genomic DNA was extracted from the blood mononuclear cell fraction using a Gentra Puregene Kit (Qiagen, Hilden, Germany) according to the manufacturer’s instructions. DNA sequencing was performed at the Sanquin Research Laboratory (Amsterdam, The Netherlands). GeneScan was used to determine the ratio between the number of exon 2 sequences of neutrophil cytosolic factor 1 (*NCF1*) gene, which encodes p47^phox^, and the number of Ψ-*NCF1* exon 2 sequences [17]. This revealed a homozygous GT deletion (c.75_76delGT) at the start of exon 2 in *NCF1*, resulting in the introduction of a frame shift and a premature stop codon (p.Tyr26HisfsTer26) predicting a truncated and inactive p47^phox^ protein.

The patient was treated with meropenem, vancomycin and liposomal amphotericin b for 2 weeks. After obtaining the results of fungal characterization, voriconazole was started and other antibiotics were removed from the treatment strategy. After 3 months, the patient had recovered as confirmed by chest imaging and clinical manifestations. The patient gained 10 kg in weight and is on maintenance treatment with voriconazole.

## Discussion and conclussions

We report here an adult autosomal CGD subject with pulmonary *A. terreus* infection*.* We are unaware of any previous cases describing pulmonary *A. terreus* infection in an adult on long-term corticosteroid treatment. We combined molecular identification of CGD mutation with fungal DNA sequencing to correctly identify the causal agent and consequently provided optimal therapy for the patient. In the first 3 months of treatment the patient gained 10 kg in weight indicating the success of the diagnosis and treatment.

Most pulmonary fungal infections in CGD patients are due to *A. fumigatus* although *A. terreus* can cause pneumonia and disseminated infections in man [18–25]. In humans, *A. fumigatus, A. flavus and A. niger* are much more common pathogens than *A. terreus* [25] although *A. terreus* has been associated with vertebral infections [26–29]*.* Immunocompromised individuals are at most risk of opportunistic infection by *A. terreus*. These patients include COPD patients on corticosteroids, cancer patients receiving chemotherapy and patients with HIV/AIDS [29–31].

It is likely, therefore that the *A. terreus* infection seen in this CGD patient with an inherited defect of PMN function and on long-term treatment with corticosteroids was opportunistic. The incidence of *A. terreus* has risen from < 2% in 1996 to 15% in 2001 [32]. The resistance of *A. terreus* to amphotericin B, thermotolerance and the production of accessory conidia have been proposed as mechanisms that explain the rapid dissemination of the organism during invasive infections [33].

*A. Terreus* is an opportunistic fungus with an increasing prevalence amongst aspergillosis species and has a higher mortality rate (51% versus 30%) compared to other species [34–37]. *A. Terreus* infection often leads to superficial infections in man whereby the inhalation of spores initiates the immediate recruitment and activation of macrophages and neutrophils that overwhelms the infection [38]. However, in immunosuppressed individuals with neutropenia, or with a compromised capacity for neutrophil phagocytosis, this response is less vigorous [39].

Most CGD patients are susceptible to opportunistic infections. Indeed, the most important symptom of this disease is recurrent and/or invasive infections, especially those caused by catalase-positive bacteria and fungi. In Western countries, about 30% of CGD cases have been reported to be autosomal recessive (AR), of which the majority is due to mutations in *NCF1* [5, 40]. However, in a recent multicenter study conducted in Iran, the majority of CGD patients were found to be AR (87%) and only a minority had X-linked CGD (13%) [41]. It is important in regions where tuberculosis is endemic, such as Iran, that sarcoidosis and CGD are considered as differential diagnosis. The demonstration of successful patient-orientated treatment after using sequencing to confirm CGD and proper microbiological methods to identify the presence of specific infectious agents emphasises the importance of adopting this approach across the region.

## Abbreviations

BAL: Bronchoalveolar lavage
CGD: Chronic granulomatous disease
DHR: Dihydrorhodamine-1,2,3
GAM: Galactomannan
NAPDH: Nicotinamide adenine dinucleotide phosphate
NBT: Nitroblue tetrazolium
NCF1: Neutrophil cytosolic factor 1
PMA: Phorbol 12-myristate 13-acetate
PMN: Polymorphonuclear neutrophils
ROS: Reactive oxygen species
